# Endometriosis as a determinant of work disability in the Hungarian population: quantifying productivity loss and the need for workplace prevention strategies

**DOI:** 10.1016/j.pmedr.2026.103501

**Published:** 2026-05-18

**Authors:** Dominika Miklos, Attila Bokor, Zsuzsanna Beretzky, Valentin Brodszky, Tamas Lakat, Gernot Hudelist, Linda Balpataki, Dora B. Balogh

**Affiliations:** a Department of Obstetrics and Gynaecology, Semmelweis University, 27 Baross Street, Budapest 1088, Hungary; b Department of Health Policy, Corvinus University of Budapest, 8 Fővám Square, Budapest 1093, Hungary; c Pediatric Centre, Semmelweis University, 53-54 Bókay János Street, Budapest 1083, Hungary; d Department of Gynaecology, Centre for Endometriosis, Hospital St. John of God, 1 Johannes-von-Gott-Platz, Wien 1020, Austria; ^d2^ Rudolfinerhaus Private Clinic and Campus, 78 Billrothstraße, Wien 1190, Austria; e Centre for Foreign Language Education and Research, Corvinus University of Budapest, 4-6 Közraktár Street, Budapest 1093, Hungary

**Keywords:** Endometriosis, Work ability, Presenteeism, Indirect costs, Occupational health, Public health

## Abstract

**Objective:**

To quantify the impact of endometriosis on work productivity and work ability and to identify modifiable workplace targets for prevention-oriented strategies.

**Methods:**

This cross-sectional study, conducted between October 2024 and January 2025 in Hungary, compared 566 women with endometriosis to 447 controls using the Work Productivity and Activity Impairment questionnaire and the Work Ability Index (WAI). Productivity loss was monetised using the human capital approach based on national average wages.

**Results:**

Women with endometriosis reported significantly higher absenteeism (9.73% vs 5.84%) and presenteeism (47.2% vs 38.4%) compared to controls. Affected individuals missed nearly twice as many work hours over four weeks (12.7 ± 31.3 vs 5.66 ± 12.6 h), resulting in an estimated annual income loss of €1757 per person. Total WAI scores were significantly lower (27.2 ± 4.48 vs 30.3 ± 4.29), with 42.0% of the endometriosis group classified in the “poor” work ability category, compared with 17.9% of controls (*p* < 0.01). Furthermore, 54% of participants reported that their employers had little to no knowledge of the condition.

**Conclusion:**

Endometriosis is a major determinant of work disability and indirect economic costs. Structured workplace accommodations represent key tertiary prevention strategies to preserve labour force participation and mitigate the socioeconomic burden of the disease.

## Highlights

• Endometriosis nearly doubles work absenteeism and significantly raises presenteeism.
• Affected women face an estimated annual income loss of approximately €1757.
• Among women with endometriosis, 42% are in the “Poor” work ability category.
• Employer awareness is low, with 54% having little to no knowledge of the condition.
• Workplace accommodation and support are essential for maintaining work capacity.

## 1 Introduction

Endometriosis is a chronic gynaecological disorder affecting approximately 10% of women of reproductive age, characterised by the presence of endometrium-like tissue outside the uterine cavity (Tomassetti et al., 2021; Becker et al., 2022). Beyond its clinical classification, it is recognised as a public health priority due to its diverse manifestations, including severe pelvic pain, dysmenorrhea, and infertility, which significantly impair daily functioning and quality of life (QoL). As a condition primarily affecting women during their peak economically active years, endometriosis carries profound implications for labour market participation and long-term socioeconomic stability (Bianconi et al., 2007; Gao et al., 2006; Giudice and Kao, 2004; Bulun, 2009).

Despite its high prevalence, the average diagnostic delay remains alarmingly long, ranging from 4 to 11 years (Sims et al., 2021). Within a preventive medicine framework, this delay represents a critical failure of secondary prevention, allowing unmanaged symptoms to progress into chronic functional limitations. The economic consequences are substantial; the total annual societal burden reaches billions of euros, with work-related indirect costs, such as absenteeism and presenteeism, constituting the largest component (Simoens et al., 2012; Pónusz-Kovács et al., 2024). In 2023, the European Parliament estimated that endometriosis-associated work absences alone generated approximately €30 billion in annual expenses (Pignedoli et al., 2023).

Prevention strategies are traditionally categorised into three levels: primary prevention aims to prevent disease onset, secondary prevention focuses on early detection and intervention to halt progression, and tertiary prevention seeks to minimize complications and preserve function in established disease.

Work productivity and work ability are critical patient-reported outcome measures PROMs that capture the real-world impact of chronic illness (Weldring and Smith, 2013). While international studies have reported substantial productivity losses, data from Central and Eastern Europe remain scarce (Nnoaham et al., 2011; Røssell et al., 2025; Rossi et al., 2021; Hansen et al., 2013; Bell et al., 2023). Furthermore, work ability is a vital intermediate indicator that links clinical morbidity to the risk of permanent labour force withdrawal. However, the role of modifiable structural determinants, such as employer awareness and workplace accommodations, has been underexplored.

To address these gaps, we conducted a large-scale comparative study in Hungary using two validated instruments: the Work Productivity and Activity Impairment (WPAI) questionnaire and the Work Ability Index (WAI) (Reilly et al., 2004; Ilmarinen, 2007). Our primary objective was to quantify the occupational and socioeconomic impact of endometriosis compared to a healthy control group and to assess long-term work ability and disability risk. Additionally, we aimed to identify modifiable targets for tertiary prevention strategies, including perceived employer support and the specific occupational adaptations required to preserve work capacity and reduce indirect societal costs.

## 2 Methods

### 2.1 Study design and population

This cross-sectional study was conducted via the Lucy mobile health (mHealth) application between October 2024 and January 2025. Participants were recruited through patient advocacy groups, healthcare providers, and targeted social media outreach campaigns. We recruited 1147 women aged 18–45 years. Each participant self-reported their medical diagnosis status regarding endometriosis. Participants completed a structured, 32-item questionnaire that gathered demographic information, work productivity and ability data, and workplace accommodations. Users provided electronic informed consent by agreeing to the privacy policy and information disclosure statement before data collection began. The data controller stored and processed only anonymised data, preserving participant confidentiality. Individuals who declined to participate in the study were excluded. Information on data protection and user agreements is accessible at https://hellolucy.app/hu/protection and https://hellolucy.app/hu/agreement. The manuscript adheres to the Strengthening the Reporting of Observational Studies in Epidemiology (STROBE) checklist for cross-sectional studies.

### 2.2 Measures

The WPAI is a validated tool designed to assess the impact of symptoms on work productivity and daily activities, measuring time being absent from work owing to symptoms (absenteeism), reduced productivity while on the job due to symptoms (presenteeism), overall work impairment (a combination of the previous two), and reduced effectiveness in non-work-related activities (activity impairment). We used the general health version of the WPAI (WPAI:GH) questionnaire (Reilly et al., 1993). The questionnaire first establishes employment status (Q1), followed by work-related questions (Q2-Q5) for employed participants, including hours missed from work due to health reasons (Q2) and other reasons (Q3), and total hours worked in the past seven days (Q4). Work productivity loss is measured on a 10-point scale from 0 (not affected) to 10 (completely prevented) (Q5). The final question (Q6) assesses how health issues impact daily activities using the same 10-point rating scale. Higher WPAI scores indicate greater impairment and reduced productivity. Scoring followed the methodology as outlined at http://www.reillyassociates.net/WPAI_Scoring.html. In addition to the standard WPAI items, participants who indicated that they had missed any hours from work due to health problems were asked to specify how many of those hours were missed specifically due to endometriosis.

To better capture symptom-related work impairment patterns in endometriosis, we used a modified 4-week recall period rather than the standard 7-day timeframe, consistent with previous studies (Nnoaham et al., 2011). This modification accounts for the cyclical nature of endometriosis symptoms and reduces potential recall bias associated with atypical symptom weeks.

All components of productivity loss (absenteeism, presenteeism) were valued applying the human capital approach using the average gross hourly wage levels (€11.53) in Hungary (Main earnings data - full circle of employers [Internet], 2025).

The WAI, developed by the Finnish Institute of Occupational Health, is a widely used instrument that assesses an individual's self-perceived capacity to perform work tasks, considering the demands of their current occupation, overall health status, and mental resources (Ilmarinen et al., 1997). The WAI comprises seven components: (1) current work ability compared to lifetime best (0–10 points); (2) work ability relative to job demands, both physical and mental (2–10 points); (3) number of current physician-diagnosed diseases (1–7 points); (4) estimated work impairment due to disease (1–6 points); (5) sick leave days in the past 12 months (1–5 points); (6) projected work ability in two years (1, 4, or 7 points); and (7) psychological resources (1–4 points). Domain scores are summed to yield a total WAI score ranging from 7 to 49, with higher scores reflecting better work ability. Scores are categorised as poor (Sims et al., 2021; Simoens et al., 2012; Pónusz-Kovács et al., 2024; Pignedoli et al., 2023; Weldring and Smith, 2013; Nnoaham et al., 2011; Røssell et al., 2025; Rossi et al., 2021; Hansen et al., 2013; Bell et al., 2023; Reilly et al., 2004; Ilmarinen, 2007; Reilly et al., 1993; Main earnings data - full circle of employers [Internet], 2025; Ilmarinen et al., 1997; Fowler et al., 2006; Hadfield et al., 1996; Husby et al., 2003; Ballard et al., 2006; Bokor et al., 2013; Fourquet et al., 2010), moderate (28–36), good (37–43), or excellent (44–49) work ability (Ilmarinen, 2007).

Free-text responses were exported to the item *“What type of workplace support or access would be most helpful for you?”* (endometriosis cohort only). Synonyms and spelling variants were harmonised with a predefined dictionary (e.g., “home office” → remote work; “hybrid work” kept as a separate label; “designated rest periods during the workday” → designated rest periods). Multi-word concepts were treated as single phrases. We computed simple term frequencies (counts per theme). We generated a word cloud with font size proportional to frequency and a fixed random seed to ensure reproducibility.

### 2.3 Statistical analysis

Descriptive statistics were calculated for continuous variables, including means, standard deviations, and 95% confidence intervals (CI). Data are presented as mean ± standard deviation (SD). Between-group comparisons for continuous variables were conducted using either an unpaired *t*-test, a Kruskal-Wallis test, or the Mann-Whitney *U* test, as appropriate, depending on the data distribution assessed by normality tests. Depending on expected cell counts, categorical variables were compared between groups using chi-square analyses and Fisher's exact tests. Statistical significance was defined as *p* < 0.05. All statistical analyses were performed using GraphPad Prism version 10.1.0 (GraphPad Software, San Diego, CA, USA).

### 2.4 Ethical approval

The study was conducted in accordance with the Declaration of Helsinki, and ethical approval was granted by the Medical Research Council of Hungary (OGYÉI/31355/2021). The study protocol is registered at ClinicalTrials.gov (Identifier: NCT06147687).

## 3 Results

### 3.1 Participant characteristics and demographics

1013 of 1147 eligible women (approximately 88.3%) consented to participate. This included 566 women with self-reported medical diagnosis of endometriosis and 477 controls who did not report an endometriosis diagnosis. As summarised in Table 1, women with endometriosis were significantly older than controls **(33.3 ± 6.13; 95% CI: 32.8**, **33.8 vs. 29.1 ± 6.77; 95% CI: 28.4**, **29.7)** and more likely to have completed higher education. Employment rates were also higher among women with endometriosis (*p* < 0.01). In the endometriosis group, the mean time from symptom onset to diagnosis was 5.01 ± 5.49 years (95% CI: 4.54, 5.49).

**Table 1:** Demographic data of adult participants in Hungary, between October 2024 and January 2025.

	Control (*n* = 447)	Endometriosis (*n* = 566)	*p* value
Age (years)	29.1 ± 6.8	33.3 ± 6.1	*p* < 0.01
Education			*p* < 0.01
Primary	32 (7.2%)	12 (2.1%)	
Secondary	210 (47.0%)	248 (43.8%)	
Higher	205 (45.9%)	306 (54.1%)	
Paid employment	345 (77.2%)	496 (87.6%)	*p* < 0.01
Time to diagnosis (years)	NA	5.0 ± 5.5	

Data are presented as mean ± SD. Statistical comparisons were performed using the Mann-Whitney U test, Kruskal-Wallis test or chi-square analyses. *p* < 0.05 vs. Control.

### 3.2 Impact of endometriosis on work productivity and daily activities

77.2% of the control group and 87.6% of individuals diagnosed with endometriosis reported paid employment (Table 1). All employed participants completed the WPAI questionnaire in full.

Women with endometriosis reported significantly greater impairments in both work productivity and daily functioning (Table 2). Absenteeism was significantly higher in the endometriosis group (9.73 ± 21.0%; 95% CI: 7.9, 11.6) than in controls (5.84 ± 14.2%; 95% CI: 4.31, 7.37). Presenteeism was also significantly elevated in women with endometriosis (47.2 ± 27.6%; 95% CI: 44.8, 49.6) compared to controls (38.4 ± 27.1%; 95% CI: 35.5, 41.2), reflecting reduced work effectiveness due to symptoms.

**Table 2:** Work productivity and ability index scores of adult participants in Hungary, between October 2024 and January 2025.

	Control (*n* = 345)	95% CI	Endometriosis (*n* = 496)	95% CI
Absenteeism (%)^a^	5.8 ± 14.2	4.31, 7.37	9.7 ± 21.0	7.9, 11.6
Presenteeism (%)^b^	38.4 ± 27.1	35.5, 41.2	47.2 ± 27.6	44.8, 49.6
Overall work impairment (%)^c^	42.1 ± 28.0	39.1, 45.1	50.8 ± 27.9	48.3, 53.3
Activity impairment (%)^d^	36.7 ± 27.4	33.8, 39.6	46.8 ± 27.5	44.4, 49.2

Data are presented as mean ± SD. Statistical comparisons were performed using the Mann-Whitney U test. p < 0.05 vs. Control. Participant numbers differed from the original sample size due to incomplete questionnaire responses.

^a^Ratio of time being absent from work owing to symptoms to total working days.

^b^Ratio of reduced effectiveness while on the job owing to symptoms to total potential productivity.

^c^Combination of absenteeism and presenteeism.

^d^Reduced effectiveness in non-work-related activities, e.g., childcare, exercise, housekeeping, etc.

Overall work impairment, combining absenteeism and presenteeism, was also significantly greater in the endometriosis group (50.8 ± 27.9%; 95% CI: 48.3, 53.3) than in controls (42.12 ± 28.04; 95% CI: 39.1, 45.1). Likewise, impairment in daily activities was more pronounced among women with endometriosis (46.87 ± 27.54%; 95% CI: 44.4, 49.2) compared with control participants (36.7 ± 27.4%; 95% CI: 33.8, 39.6).

Over a four-week period, respondents with endometriosis missed an average of 12.7 ± 31.3 h (95% CI: 9.96, 15.5) of work due to health-related issues. In contrast, individuals in the control group reported an average of 5.7 ± 12.6 h (95% CI: 4.33, 6.99) of work missed.

### 3.3 Work ability index comparisons between the endometriosis and control groups

Based on the analysis of data from respondents both with and without current paid employment, we observed that participants in the endometriosis group demonstrated significantly lower total WAI scores compared with controls (27.2 ± 4.48; 95% CI: 26.8, 27.6 vs. 30.3 ± 4.29; 95% CI: 29.9, 30.7;). When examining specific components, the endometriosis group also showed a lower rating of current work ability compared to their lifetime best (6.19 ± 2.25; 95% CI: 6.01, 6.37 vs. 6.64 ± 2.29; 95% CI: 6.43, 6–85;) and reduced mental resource scores (6.68 ± 2.47; 95% CI: 6.48, 6.88 vs. 7.26 ± 2.58; 95% CI: 7.03,7.50;). These findings indicate a pronounced negative impact of endometriosis on overall work ability and mental well-being (Table 3).

**Table 3:** Work ability index scores of adult participants in Hungary, between October 2024 and January 2025.

	Control (*n* = 392)	95% CI	Endometriosis (*n* = 555)	95% CI
Total score	30.3 ± 4.3	29.9, 30.7	27.2 ± 4.5	26.8, 27.6
Current work ability compared with the lifetime best	6.6 ± 2.3	6.43, 6.85	6.2 ± 2.2	6.01, 6.37
Mental resource scores	7.3 ± 2.6	7.03, 7.50	6.7 ± 2.5	6.48, 6.88

Data are presented as mean ± SD. Statistical comparisons were performed using the Mann-Whitney *U* test. *p* < 0.05 vs. Control. Participant numbers differed from the original sample size due to incomplete questionnaire responses.

In the endometriosis group, 42.0% of participants fell into the “Poor” work ability category. In contrast, only 17.9% of controls were classified as “Poor.” While 57.8% of the endometriosis cohort and 78.3% of controls fall into the “Moderate” range, only a small fraction of participants in either group attain “Good” work ability, and none reach “Excellent” (Table 4).

**Table 4:** Distribution of Work Ability Index (WAI) scores by category in control and endometriosis groups of adult participants in Hungary, between October 2024 and January 2025.

Total Score	Work Ability Category	Interpretation	Control (n = 392)	Endometriosis (n = 555)
7–27	**Poor**	High risk of work disability, urgent intervention needed	70 (17.9%)	233 (42.0%)
28–36	**Moderate**	Work ability is reduced, workplace support needed	307 (78.3%)	321 (57.8%)
37–43	**Good**	Can continue working with minor adjustments	15 (3.8%)	1 (0.2%)
44–49	**Excellent**	No limitations in work ability	0	0

Data are presented as the number of participants (percentage of the group). Participant numbers differed from the original sample size due to incomplete questionnaire responses.

A Fisher's exact test confirms a highly significant difference in the distribution of WAI categories between the two groups (*p* < 0.0001).

### 3.4 Endometriosis in the workplace: employee experiences of support and awareness

To assess workplace experiences, women with endometriosis were surveyed about their ability to communicate about their condition, perceived employer support, and desired accommodations. When asked whether women with endometriosis could discuss their condition with their employer or supervisor, most participants (67.5%, *n* = 382) reported feeling comfortable having these conversations. Approximately one-third (32.5%, *n* = 184) indicated they could not discuss it at work.

Participants were asked to rate their workplace's general support for women's health issues, including endometriosis. Our findings showed that while 39.9% of women perceived their workplace as supportive or very supportive of women's health issues, a substantial proportion (39.8%) remained neutral, and 20.4% reported inadequate support.

When specifically asked about their employer's knowledge and understanding of endometriosis, only 17% of participants believed their workplace had a good or excellent understanding of the condition, while over half (54%) reported that their employer had little to no knowledge of the condition.

**Fig. 1:**
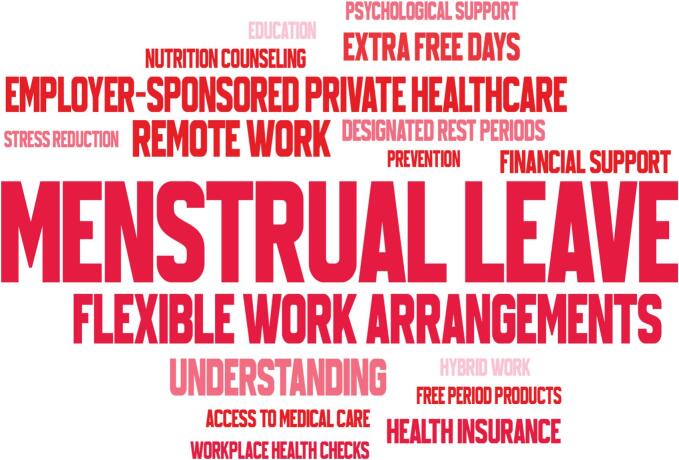
Requested workplace supports among women with endometriosis in Hungary, between October 2024 and January 2025 (free-text responses). Word cloud of the same themes, with font size proportional to frequency.

When asked about other types of workplace support that would be beneficial, participants most frequently mentioned menstrual leave, flexible work arrangements, employer-sponsored private healthcare, remote work, employer understanding, and financial support (Fig. 1). These responses emphasise the need for comprehensive workplace policies that acknowledge the chronic and unpredictable nature of endometriosis symptoms.

## 4 Discussion

This study used validated WPAI and WAI instruments to characterise the occupational burden associated with endometriosis in the Hungarian population. We analysed data collected from 1013 women with self-reported endometriosis and controls using a four-week reference period (Nnoaham et al., 2011; Fowler et al., 2006). Our findings reveal that endometriosis creates a multifaceted challenge in occupational settings, affecting not only individual work performance but also exposing systemic deficiencies in workplace accommodation and understanding of women's health conditions.

Productivity analysis showed striking differences between women with self-reported endometriosis and controls across all measured domains. Affected women demonstrated nearly double the absenteeism and significantly higher presenteeism, resulting in overall work impairment. Participants with endometriosis reported missing nearly twice as much work time over a four-week period.

Based on current average earnings in Hungary, the observed work time loss corresponds to an estimated annual income loss of approximately €1757 per affected individual. Members of the control group experienced a significantly lower loss, averaging €784 per year. This conservative estimate underscores the need for employer-led health promotion programs to mitigate indirect societal costs. While the nominal loss is lower than the European Union average (€6298) due to Hungary's lower wage levels, the relative productivity impact is comparable to that of international reports (Simoens et al., 2012). Importantly, this conservative estimate excludes the considerable burden of presenteeism and does not account for direct medical expenses.

In our cohort, women with endometriosis demonstrated worse work ability than controls, with lower total WAI scores and a striking concentration in the ‘Poor’ category (42.0% vs 17.9%). As the WAI integrates longer-horizon capacity to meet job demands, this distribution indicates elevated risk of work disability and erosion of sustained labour force participation.

Our data show that, although two-thirds of women with endometriosis can discuss their condition at work, perceived organisational support remains modest, and employer knowledge is limited (only ∼17% report a good/excellent understanding). Free-text analysis identified key accommodation needs, including menstrual leave, flexible working arrangements, remote/hybrid working, understanding, protected rest periods, paid medical appointments, employer-sponsored healthcare, and psychological support.

Based on data from the endometriosis group, the mean time to diagnosis was 5.01 ± 5.49 years, shorter than international estimates and consistent with previously published data from Hungary (Nnoaham et al., 2011; Hadfield et al., 1996; Husby et al., 2003; Ballard et al., 2006; Bokor et al., 2013). This implies that symptoms often begin years before diagnosis, frequently overlapping with schooling and early career, and may have plausible downstream effects on education and employment. Despite increased awareness and guideline updates, diagnostic delay has not improved over the past decade, underscoring the urgent need for system-level interventions.

Strengths include a large, diverse mHealth sample, a control group, the use of validated instruments, and concurrent reporting of WAI totals and categories. The app-based recruitment strategy enabled efficient, scalable enrolment across working-age women. This design addresses limitations in prior studies (small samples, case-only designs, and limited use of validated measures). An important limitation is our reliance on self-reported diagnosis without verification of diagnostic modality. While participants confirmed having a medical diagnosis of endometriosis, we did not explicitly ascertain whether the diagnosis was surgical or imaging-based, which may have introduced misclassification bias. Other limitations include mHealth sampling (selection/recall bias) and restricting WPAI work-domain analyses to currently employed participants, which may underestimate population-level impact if some women left the workforce due to symptoms. Additionally, this study utilised unadjusted analyses that did not control for potential confounding variables (e.g., age), which differed between groups. Generalisability may be limited by the Hungarian labour market and social insurance context, as well as by employer leave and remote work policies. A further limitation is the lack of assessment of symptom severity and its potential impact on work ability and productivity. Such investigations should represent an important area for future research.

To our knowledge, no large-scale study has previously examined work performance and workplace accommodations among patients with endometriosis in Central and Eastern Europe.

Our findings align with international studies demonstrating a significant association between endometriosis and impairments in occupational functioning. This is reflected in increased work absenteeism, reduced on-the-job efficiency, and greater disruption of routine daily activities (Røssell et al., 2025; Rossi et al., 2021; Hansen et al., 2013; Bell et al., 2023; Fourquet et al., 2010; Soliman et al., 2017). However, most previous studies employed case-only designs without control groups. While a matched case-control study from Switzerland, Germany, and Austria documented worse work-related outcomes among women with surgically confirmed endometriosis, it did not utilise standardised productivity measures such as the WPAI or WAI (Sperschneider et al., 2019).

Only a limited number of studies have assessed work ability in endometriosis using the WAI with appropriate control groups. Our results align with prior comparative studies. Population-based data from Finland show that endometriosis is associated with poorer work ability and more disability days at midlife (Rossi et al., 2021). Danish data associate endometriosis with lower WAI and greater sick leave (Hansen et al., 2013). An Australian community study of working women reported nearly doubled odds of poor-to-moderate work ability among women with endometriosis after multivariable adjustment (Bell et al., 2023).

Our results on necessary workplace accommodations align with small-cohort qualitative studies, in which recorded discussions emphasise the need for supportive employers and flexibility to take time off for symptoms and treatment (Sperschneider et al., 2019; Moradi et al., 2014; Denny, 2004; Gilmour et al., 2008). The Australian Endo@Work project includes employer-focused work to co-design endometriosis-inclusive workplace guidance, suggesting leadership buy-in, flexible work arrangements, and education (Howe et al., 2025). The convergence across methods, including our survey data, employee interviews, and employer-focused qualitative work, provides a multi-perspective rationale for targeted workplace strategies. However, prospective evaluations are still needed to quantify the impact of these accommodations on work ability and productivity.

The convergence of our data with international findings suggests that structured workplace adaptations should be viewed as essential tertiary prevention strategies. Such interventions are not merely accommodations but economic investments in preserving the productivity of a significant portion of the female workforce.

## 5 Conclusions

Endometriosis is associated with substantial impairment in work productivity and work ability, identifying occupational functioning as a critical intermediate outcome that links chronic morbidity to long-term socioeconomic risk. The high prevalence of poor work ability among affected women indicates increased vulnerability to sustained labour force detachment. Within a preventive medicine framework, limited workplace accommodation represents a modifiable determinant of functional decline. Structured occupational adaptations, such as flexible work arrangements and employer education, function as tertiary prevention to preserve work capacity and mitigate indirect societal costs. Addressing these factors is essential to reducing avoidable productivity losses and protecting women's economic participation during their peak working years.

## CRediT authorship contribution statement

**Dominika Miklos:** Writing – original draft. **Attila Bokor:** Writing – review & editing, Conceptualization. **Zsuzsanna Beretzky:** Writing – review & editing, Formal analysis. **Valentin Brodszky:** Writing – review & editing, Formal analysis. **Tamas Lakat:** Visualization, Formal analysis. **Gernot Hudelist:** Writing – review & editing. **Linda Balpataki:** Writing – review & editing. **Dora B. Balogh:** Writing – original draft, Supervision, Methodology, Conceptualization.

## Funding sources

This study was funded by the Finding Endometriosis using Machine Learning (FEMaLe) project, funded by the European Union's Horizon 2020 Research and Innovation Programme (grant number 101017562) and by the János Bolyai Research Scholarship of the Hungarian Academy of Sciences (BO/00147/25/5).

## Declaration of competing interest

The authors declare the following financial interests/personal relationships which may be considered as potential competing interests: Attila Bokor reports financial support was provided by European Union’s Horizon 2020 Research and Innovation Programme. Dora B. Balogh reports financial support was provided by János Bolyai Research Scholarship of the Hungarian Academy of Sciences. If there are other authors, they declare that they have no known competing financial interests or personal relationships that could have appeared to influence the work reported in this paper.

## Data Availability

Data will be made available on request.

## References

[bb0005] Ballard K., Lowton K., Wright J. (2006). What’s the delay? A qualitative study of women’s experiences of reaching a diagnosis of endometriosis. Fertil. Steril..

[bb0010] Becker C.M., Bokor A., Heikinheimo O., Horne A., Jansen F., Kiesel L. (2022). ESHRE guideline: endometriosis. Hum. Reprod. Open.

[bb0015] Bell R.J., Robinson P.J., Skiba M.A., Islam R.M., Hemachandra C., Davis S.R. (2023). The impact of endometriosis on work ability in young Australian women. Aust. N. Z. J. Obstet. Gynaecol..

[bb0020] Bianconi L, Hummelshoj L, Coccia ME, Vigano P, Vittori G, Veit J, et al. Recognizing endometriosis as a social disease: the European Union-encouraged Italian Senate approach. Fertil. Steril. 88. United States, 2007. p. 1285–7. 10.1016/j.fertnstert.2007.07.1324 17991515

[bb0025] Bokor A., Koszorús E., Brodszky V., D’Hooghe T., Rigó J. (2013). The impact of endometriosis on the quality of life in Hungary. Orv. Hetil..

[bb0030] Bulun S.E. (2009). Endometriosis. N. Engl. J. Med..

[bb0035] Denny E. (2004). Women’s experience of endometriosis. J. Adv. Nurs..

[bb0040] Fourquet J., Gao X., Zavala D., Orengo J.C., Abac S., Ruiz A. (2010). Patients’ report on how endometriosis affects health, work, and daily life. Fertil. Steril..

[bb0045] Fowler J.F., Ghosh A., Sung J., Emani S., Chang J., Den E. (2006). Impact of chronic hand dermatitis on quality of life, work productivity, activity impairment, and medical costs. J. Am. Acad. Dermatol..

[bb0050] Gao X., Yeh Y.C., Outley J., Simon J., Botteman M., Spalding J. (2006). Health-related quality of life burden of women with endometriosis: a literature review. Curr. Med. Res. Opin..

[bb0055] Gilmour J.A., Huntington A., Wilson H.V. (2008). The impact of endometriosis on work and social participation. Int. J. Nurs. Pract..

[bb0060] Giudice L.C., Kao L.C. (2004). Endometriosis. Lancet.

[bb0065] Hadfield R., Mardon H., Barlow D., Kennedy S. (1996). Delay in the diagnosis of endometriosis: a survey of women from the USA and the UK. Hum. Reprod..

[bb0070] Hansen K.E., Kesmodel U.S., Baldursson E.B., Schultz R., Forman A. (2013). The influence of endometriosis-related symptoms on work life and work ability: a study of Danish endometriosis patients in employment. Eur. J. Obstet. Gynecol. Reprod. Biol..

[bb0075] Howe D., O’Shea M., Duffy S., Armour M. (2025). From insight into action: understanding how employer perspectives shape endometriosis-inclusive workplace policies. Healthcare (Basel).

[bb0080] Husby G.K., Haugen R.S., Moen M.H. (2003). Diagnostic delay in women with pain and endometriosis. Acta Obstet. Gynecol. Scand..

[bb0085] Ilmarinen J. (2007). The Work Ability Index (WAI). Occup. Med..

[bb0090] Ilmarinen J., Tuomi K., Klockars M. (1997). Changes in the work ability of active employees over an 11-year period. Scand. J. Work Environ. Health.

[bb0095] Main earnings data - full circle of employers [Internet]. 2025.05.20. [cited 2025.08.21.]. Available from: https://www.ksh.hu/stadat_files/mun/en/mun0046.html.

[bb0100] Moradi M., Parker M., Sneddon A., Lopez V., Ellwood D. (2014). Impact of endometriosis on women’s lives: a qualitative study. BMC Womens Health.

[bb0105] Nnoaham KE, Hummelshoj L, Webster P, d'Hooghe T, de Cicco Nardone F, de Cicco Nardone C, et al. Impact of endometriosis on quality of life and work productivity: a multicenter study across ten countries. Fertil. Steril. 2011;96(2):366–73.e8. 10.1016/j.fertnstert.2011.05.090 PMC3679489 21718982

[bb0110] Pignedoli S, Chastel O, Gregorová M, Meo SD, Motreanu D-Ş, Donato F, et al. Current state of the fight against endometriosis in the EU 2023 [updated 2023.02.15.

[bb0115] Pónusz-Kovács D., Pónusz R., Sántics-Kajos L.F., Csákvári T., Kovács B., Várnagy Á. (2024). Evaluation of the epidemiological disease burden and Nationwide cost of endometriosis in Hungary. Healthcare (Basel)..

[bb0120] Reilly M.C., Zbrozek A.S., Dukes E.M. (1993). The validity and reproducibility of a work productivity and activity impairment instrument. Pharmacoeconomics.

[bb0125] Reilly M.C., Bracco A., Ricci J.F., Santoro J., Stevens T. (2004). The validity and accuracy of the Work Productivity and Activity Impairment questionnaire--Irritable Bowel Syndrome version (WPAI:IBS). Aliment. Pharmacol. Ther..

[bb0130] Røssell E.L., Plana-Ripoll O., Josiasen M., Hansen K.E., Bech B.H., Rytter D. (2025). Association between endometriosis and working life among Danish women. Hum. Reprod..

[bb0135] Rossi H.R., Uimari O., Arffman R., Vaaramo E., Kujanpää L., Ala-Mursula L. (2021). The association of endometriosis with work ability and work life participation in late forties and lifelong disability retirement up till age 52: a northern Finland birth cohort 1966 study. Acta Obstet. Gynecol. Scand..

[bb0140] Simoens S., Dunselman G., Dirksen C., Hummelshoj L., Bokor A., Brandes I. (2012). The burden of endometriosis: costs and quality of life of women with endometriosis and treated in referral centres. Hum. Reprod..

[bb0145] Sims O.T., Gupta J., Missmer S.A., Aninye I.O. (2021). Stigma and endometriosis: a brief overview and recommendations to improve psychosocial well-being and diagnostic delay. Int. J. Environ. Res. Public Health.

[bb0150] Soliman A.M., Coyne K.S., Gries K.S., Castelli-Haley J., Snabes M.C., Surrey E.S. (2017). The effect of endometriosis symptoms on absenteeism and Presenteeism in the workplace and at home. J. Manag. Care Spec. Pharm..

[bb0155] Sperschneider M.L., Hengartner M.P., Kohl-Schwartz A., Geraedts K., Rauchfuss M., Woelfler M.M. (2019). Does endometriosis affect professional life? A matched case-control study in Switzerland, Germany and Austria. BMJ Open.

[bb0160] Tomassetti C., Johnson N.P., Petrozza J., Abrao M.S., Einarsson J.I., Horne A.W. (2021). An international terminology for endometriosis, 2021. J. Minim. Invasive Gynecol..

[bb0165] Weldring T., Smith S.M. (2013). Patient-reported outcomes (PROs) and patient-reported outcome measures (PROMs). Health Serv Insights..

