# Supplementary material for: Endometriosis as a determinant of work disability in the Hungarian population: quantifying productivity loss and the need for workplace prevention strategies
Source: Prev Med Rep. 2026 May 18;66:103501. doi: 10.1016/j.pmedr.2026.103501 (PMC13213222; doi:10.1016/j.pmedr.2026.103501)
Supplement: Supplementary material 1 [file mmc1.docx]

**SUPPLEMENTARY DATA**

**Supplemental Table S1.** Work ability index scores. Data are presented as mean ± SD.

|  | Control | 95% CI | Endometriosis | 95% CI | *p* value |
| --- | --- | --- | --- | --- | --- |
| Total score | 30.3 ± 4.29 | 29.9–30.7 | 27.2 ± 4.48 | 26.8–27.6 | *p* <0.0001 |
| Current work ability compared  with the lifetime best | 6.64 ± 2.29 | 6.43–6.85 | 6.19 ± 2.25 | 6.01–6.37 | *p* < 0.0003 |
| Mental resource scores | 7.26 ± 2.58 | 7.03–7.50 | 6.68 ± 2.47 | 6.48–6.88 | *p* < 0.0008 |

Statistical comparisons were performed using the Mann-Whitney U test. *p* < 0.05 *vs.* Control
